# The asymmetric effects of institutional quality on financial inclusion in the Asia-pacific region

**DOI:** 10.1016/j.heliyon.2022.e12016

**Published:** 2022-11-30

**Authors:** Loan Thi-Hong Van, Nhan Thien Nguyen, Hung Le-Phuc Nguyen, Duc Hong Vo

**Affiliations:** a School of Advanced Study, Ho Chi Minh City Open University, Viet Nam; b The Research Centre in Business, Economics and Resources, Ho Chi Minh City Open University, Viet Nam

**Keywords:** Institutional quality, Financial inclusion, Asymmetric effects, Panel smooth transition regression, Asia-pacific region

## Abstract

Financial inclusions are generally considered an effective mechanism to support sustainable economic growth in emerging markets. While the symmetric effects of institutional quality on financial inclusion have been widely investigated, their asymmetric effects have largely been ignored in existing literature, particularly for emerging markets. In this paper, we estimate the index of financial inclusion for 19 countries in the Asia-Pacific region from 2004 to 2020. The institutional quality is proxied by five indicators, including (i) business sophistication, (ii) regulatory quality, (iii) investment freedom, (iv) government effectiveness, and (v) the rule of law. The advanced panel smooth transition technique ensures that the asymmetric effects of institutional quality on financial inclusion are substantiated depending on the income level across countries in the sample. We find that institutional quality's effects on financial inclusion are asymmetric depending on the income level. Our findings indicate that middle-income countries such as Vietnam and other emerging nations in the Asia-Pacific region mostly benefit from the positive effects of institutional reform to ensure more inclusive economic growth in the future.

## 1 Introduction

The pioneering studies from Schumpeter (1911) and later of McKinnon (1973) have shifted views of finance on the economy. Previous studies consider that economic growth is a function of only physical and human capital. Finance is not involved. However, this perception, generally known as the demand-following hypothesis, gradually loses ground. The financial sector facilitates the accumulation of capital and advances the total factor productivity embedded in the flows of foreign direct investment (FDI). The modern economy lays the foundation for the emergence of financial inclusion, which determines the state of the economy and the society in which every economic entity has access to the financial services provided at affordable prices and in a sustainable manner.

A wide range of academic studies has provided evidence on the beneficial effects of financial inclusion on various aspects of the socio-economical issues, such as eradicating poverty, achieving sustainable economic growth, assuring the inclusiveness of government policies, or further rendering resilience to the financial sector. In detail, many studies find that access to financial services, low-income households, and small and middle enterprises (SMEs) will significantly promote their future consumption and the accessibility to financing activities (Kim et al., 2017; Adomako et al., 2016; Sarma and Pais, 2011). Participation in the financial market also positively affects disadvantaged households' well-being (Bookwalter and Dalenberg, 2010; Li, 2018). Financial inclusion also improves the allocation of resources from the government's pro-poor programs and reduces the cost of waiting, travelling and possibly bribing for receiving social assistance once the transfers are digitalized (Aker et al., 2016; Muralidharan et al., 2014). It is also well-established that financial inclusion provides banks with low-cost and stable sources of deposit, which contribute to a strong base for lending activities. The low-income savers and borrowers also keep their financial activities, including deposits and borrowing, unchanged (Vo et al., 2020).

Over the last decade, promoting financial inclusion is clearly an ultimate goal to achieve all-inclusive growth for many countries, especially developing nations. For example, in the United States, the Community Reinvestment Act was enacted in late 1977 to cover credit shortfall to low- and middle-income households. As a result, people with credit discrimination can now borrow from financial institutions in compliance with safe and sound operations. For India, the Reserve Bank of India (RBI) initiates courses of action that aim to enhance the inclusiveness of the financial sector. These initiatives include (i) loosening constraints for the account opening and lending process, (ii) allowing commercial banks to open financial hubs from Tier 2 to Tier 6 freely, and (iii) advising banks to provide the Basic Saving Bank Deposit accounts, which serve the primary facilities such as depositing, withdrawing, and wiring money. However, it is observed that while financial reforms in favour of financial inclusion have been successfully implemented in one country, they may be appropriate in the others. Table 1 presents the attempts implemented by many governments globally to boost the level of financial inclusion and their corresponding outcomes.

**Table 1:** Policies and achievements on financial inclusion in selected countries.

Country	Policies	What they do	How effective it is
The USA	Community Reinvestment Act (CRA)	- Allowing those whom banks have rejected to have a chance to borrow. - Financial institutions have the right to choose whether or not to follow the Act.	- The percentage of credit to CRA-covered households increases sharply.
France	Law on exclusion (1998). Right to the Basic account (2013).	- Assuring that all citizens are provided with at least one bank account with basic functions.	- High bank account coverage but lack of access to finance. The borrowing rate at the age of +15 was less than 20 per cent in 2017.
India	Financial Sector Reform (2009) Committee on Financial inclusion (2006)	- Providing basic accounts for its citizens, such as savings, small credit, overdraft, and receiving and transferring money (Sarma and Pais, 2011; Nair, 2017). - Enhancing competition in the banking sector. Categorizing banks according to their targeted clients and scope of activities (GoI, 2009)	- Bank branches in the rural areas increased by nine-fold, from 67,694 in 2010 to 553,713 in 2015. - Money transferred by the program increases by 41 per cent. - The financial utilization remains low among newly created accounts. On the other hand, India has one of the highest bank account rates.
South Africa	Financial Sector Charter (2003)	- Accessing financial services is fundamental to economic growth. - Ensuring poor households, SMEs and other financially disadvantaged groups are targeted by financial institutions. - Requiring financial institutions to spend at least 0.2 per cent Earning After Tax (EAT) on financial literacy.	- The African banking sector is one of the most liquid sectors globally (Honohan and Beck, 2007). - The declining trend in credit to private sectors (Allen et al., 2010).
China	Plan for advancing the development of financial inclusion (Chen and Yuan, 2021)	- Promoting customer satisfaction, availability, and accessibility of financial services. - Implementing the "Three rural" reduced the required reserve for the bank that had met the targeted credit for marginalized segments. - Earmarking funds and tax exemption for state-owned firms and financial institutions lower the credit risk of catering financial services to the “unbanked” groups.	- Credit provided by financial sectors to the agriculture and SMEs accounts for 26 per cent and 28 per cent of the refinancing amount.
Vietnam	Government's Decision to approve the national financial inclusion program	- Proposed multiple targets that needed to be met by financial sectors before 2025. - Allowing non-bank entities to provide basic banking services. - Estimating affordable banking fees. - Encouraging economic entities to participate in microlending.	- Both the number and value of online payments increase significantly. - The coverage of credit information centres accounts for 60 per cent of adults. - The financial infrastructure in rural areas, such as ATMs, or bank outlets, has yet to be developed.

The outcomes of some programs are not as desired. The existing literature describes financial inclusion as a multifaceted phenomenon. It is more likely an outcome of the market adjustment process where financial provision and usage are at equilibrium. For example, studies by Samar and Pais (2011), Van et al. (2019), and Karim et al. (2021) discuss that the degree of financial inclusiveness is determined by three distinct dimensions, which are accessibility, availability, and usage of banking services.

The effect of institutional quality on the depth of the financial sector has recently become a much-debated topic. However, theoretical consideration and empirical evidence are inconsistent. Two approaches are currently used to examine the extent to which the quality of the environment in which banks operate influences the financial accessibility of financially deprived economic entities. The first approach is that financial statements of banks or firms and macroeconomic indicators are used to explain the decisions of providing financial services or engaging in a lending relationship with formal financial institutions. Findings from those studies confirm that the environmental factors cause the lack of access to financial services and the inefficient financial allocation (Ahamed and Mallick, 2019). For example, Ezirim et al. (2004), Love and Peria (2012), Andrianova et al. (2015), and Zulkhibri and Ghazal (2017) consider that both the lack of credit information centres and poor regulatory practice make it harder for the financial sector to efficiently allocate economic resources because the cost associated to screening and building lending relationship is difficult to internalize.

The other approach uses only macroeconomic indicators to examine the extent and direction that institutional factors affect the country-wise market-defined financial inclusion. However, this strand of research remains relatively limited because the benefits of financial inclusion attract much attention compared to how to improve financial inclusion (Kochhar, 2010). Talmaciu (2014), and Hechmy (2016) argue that financial development is more of a problem of institutional factors than a mismatch of supply and demand. They also consider that the quality of financial superstructure should precede or at least attain a certain threshold before the financial development. On that basis, recent studies by Muriu (2020) and Eldomiaty et al. (2020) consider that the geographical impact of institutional infrastructure is important. However, the index used as a proxy for financial inclusion does not reflect this important aspect of institutional infrastructure. Specifically, the financial inclusion index used in their analysis is from the Global Findex, a single-dimension indicator, representing a “number of depositors with commercial banks” as in the former study.

The contributions of this paper to the existing literature on financial inclusion are twofold. *First*, our analysis develops the new financial inclusion index from 2004 to 2020. Our index considers fundamental aspects of financial inclusion to establish a composite and comparable measure while eliminating the effect of correlation between its components. *Second*, we use the panel smooth transition regression (PSTR), first introduced by Gonzalez et al. (2004), to investigate the asymmetric effect of institutional factors on financial inclusion at different income levels. As discussed by Karikari (2010), and Hechmy (2016), the environment in which banks and other financial institutions operate exerts a proportional impact on the financial sector. Specifically, the institutional factors of the African countries should attain a certain level of development to make their financial sector flourishes. On that premise, our analysis differs from other studies by considering the outer environment to income level rather than conditioning it to the geographical and cultural factors. It is more insightful that higher-income countries probably have more integrated and supporting legislative systems while lower-income countries have not.

Following this introduction, the remainder of the paper is structured as follows. Section 2 discusses and synthesizes relevant studies on institutional quality and financial inclusion. Section 3 of the paper presents a research methodology. Empirical findings are then presented and discussed in section 4, followed by section 5 on the main conclusions and policy implications.

## 2 Literature review on institutional quality and financial inclusion

Both financial inclusion and institutional quality are multifaceted and abstract concepts. Scholars often utilize the adults' possibilities of participation in the financial market as a proxy for the extent of financial inclusion. In addition, scholars link financial inclusion to the macroeconomic environment in which the financial system functions are optimally facilitated and carried out (Cihak et al., 2013). These functions include (1) optimizing and facilitating the reallocation of resources, (2) spurring trade, (3) exerting the corporate governance on firms who funnel resources through the financial market, and (4) acting as a tool for diluting risks. This section discusses the theoretical aspect of the relationship between institutional quality and financial market functionality. We begin with these works on the law and finance theory of La Porta et al. (1997, 1998) and Demirguc-Kunt and Maksimovic (1998), which assume that financial development significantly depends on the nature and quality of the judicial systems. Economic entities are encouraged to engage in financial activities, especially for small and middle enterprises or poor households. The effectiveness of the institutions also relates to access to financial services such as deposits or loans. Better institutional quality lessens the amount of laborious and unnecessary procedures in such activities and ties closely to financial inclusion. The theory of institutional quality contains three broad categories in economics, culture and politics (La Porta et al., 1999). These broad categories concerning finance and their role in promoting financial inclusion are discussed below.

First, many papers share the same research interest on the nexus between financial inclusion and institutional effectiveness. An effective government from the non-interventionists perspective theoretically comes with a decent regulation, an effective tax system and a relatively high degree of marketization. For example, McKinnon (1973) and Shaw (1973) pointed to the role of trade liberalization in enriching household savings, which are considered significant constituents of the total fund for lending activities. Moreover, they also argue that the constraints on stipulating the upper and lower bound of interest often adhere to the decrease in deposits base and the interest rate. However, the countervailing arguments discussed by Galor and Zeira (1993) and Banerjee and Newman (1993). These studies highlight the substantive role of regulatory restrictions in providing the accessibility of people (especially the poor) to multiple financing channels. These studies also assume that borrowing is costly and challenging to the agents in the presence of market imperfection. As a result, these agents consider altering their current occupational direction by enriching their accumulated human capital. However, they have not had sufficient wealth as required as collaterals. Moreover, poor households are discouraged from financial activities because of high administrative and tracking costs, which are mainly ascribed to the common monopolistic nature of banks.

Second, concerning trade liberalization and financial deregulation, the mechanism through which the efficacy of a legal framework and financial inclusion interacts is now considered. The nexus has also received attention from various researchers over decades. We begin with the works of La Porta et al. (1998) and Rajan and Zingales (1998), which support the impact of different families on investors' willingness to participate in the capital markets. These studies examine the nexus between accounting standards, law enforcement, and the development of capital-intensive sectors. The capital structure of these sectors relies heavily on external financing sources. Countervailing arguments on the central role of a legal system in either establishing investor protection laws or facilitating the development of the financial market are found in Roe's (1994) and Pagano and Volpin's (2001) analyses. In these studies, the authors assume that the political economy is not the exogenous variable in determining the degree of investor protection.

The third aspect of institutional quality is the relationship between fraudulent activities and their social cost (La Porta et al., 1999). The high-income countries probably have a much more effective financial system, and their citizens have a high degree of financial awareness. North (1990) considered that the magnitude of the economic activities could explain the performance of governments. Since the scale of any specific economy is known as an endogenous variable, this assumption hardly reflects the practice that there are some small, emerging markets with distinctive institutional quality. Zulkhibri and Ghazal (2017) argued that the demand for financial services is fundamental to modern life and irrespective of income. Using the Bayesian VAR model, Dahiya and Kurma (2020) confirmed that the usage dimension of financial inclusion directly links with economic growth.

In conclusion, both financial inclusion and institutional quality are broad economic concepts. They might affect each other through various mechanisms. Our literature review indicates the necessity for our study to be conducted to examine the effect of institutional quality on financial inclusion in the Asia-Pacific region. In particular, this study focuses on the asymmetric effects of institutional quality on financial inclusion, which have been largely ignored in the existing literature.

## 3 Data and method

### 3.1 The new measurement of financial inclusion

Two strands of research differ in estimating the financial inclusion index. These two approaches are generally known as the non-parametric and parametric approaches. Concerning the non-parametric approach, Sarma (2008) constructed the index, which has received attention from various scholars such as Van et al. (2019) and Dahiya and Kumar (2020). In this measurement, the degree of financial inclusion is numerically represented by the Euclidian distance from the country's current state to the ideal point I(1,1,1,…,1). All indicators, which are used as the inputs for the calculations, are normalized. As a result, the index receives only values from 0 to 1. This approach exhibits two significant disadvantages, including (i) the correlation among the sub-indices that are used to capture the multidimensionality of the financial inclusion and (ii) the exogenous choices of weight for each dimension (Camara and Tuesta, 2014).

Concerning the parametric approach, Ahamed and Mallik (2019) argued that financial inclusion contains two dimensions, namely (i) financial outreach, which denotes the permeability of the financial system and financial usage, and (ii) accessibility to formal financial services. By Camara and Tuesta (2014), Tram et al. (2021), and Avom et al. (2021) analysis, the authors also measure financial inclusion by sequentially applying the principal component analysis (PCA) to two dimensions of financial inclusion. However, our study is different from these analyses. Instead of taking certain principal components to capture a predetermined level of variations, previous studies only retain the components whose eigenvalue is greater than one. We consider that doing so may not be appropriate because if the variance and covariance matrix are unknown, taking the eigenvectors whose eigenvalues are greater than one will be misleading in estimating the financial inclusion index.

Based on the weaknesses of previous approaches, in this paper, we develop a new index of financial inclusion that can be used to measure the degree of financial inclusion on a yearly basis. In our index, two dimensions are considered, including (i) financial usage and (ii) financial outreach. For example, Beck et al. (2007) and Allen et al. (2014) considered that the distance to financial access points appears to be an obstacle to deepening the financial system. We utilize two indicators as a proxy for the financial penetration: (i) the number of commercial bank branches per 100,000 adults; and (ii) the number of ATMs per 100,000 adults. These indicators are collected annually from the financial access survey (FAS) from 2004 to 2017. Similarly, financial usage is proxied by the number of credit cards per 1,000 adults and the number of debit cards per 1,000 adults.

The choice of our variables reflects the tendency to use banking products on the demand side and the overhead costs to promote the financial depth on the supply side. Thanks to technological advancement, we now have many apps that carry out limited functions of financial institutions such as money wiring, purchasing online, or even filing loan applications. In other words, we do not need to approach bank branches to conduct traditional transactions. Therefore, the proxies for the supply-side of financial inclusion are impractical. The same reasoning can also be considered concerning proxies on the demand side. For example, people appear to use online payment applications integrated into their mobile phones instead of cards or cash. Demirguc-Kunt et al. (2017) and Vo and Nguyen (2021) argue that financial technology conditions the payment behaviours of both financial users and non-bank organizations such as grocery stores. It is not likely to increase or decrease the number of accounts at formal financial institutions since the mobile money account must be linked with the bank accounts to ameliorate the risk of money laundering. Baharas et al. (2020) showed that countries with a higher degree of formal financial usage, such as Singapore, do not necessarily imply that the country has achieved a significant base of mobile money usage. For example, Kenya has roughly 70 per cent of adults with mobile money accounts, but only 40 per cent of adults have bank accounts. In contrast, Singapore has over 90 per cent of adults joining the banking system, while only 10 per cent have a mobile money account (Barajas et al., 2020). As such, we consider that our proxies for financial inclusion are appropriate.

We consider that the indicators used as a proxy for each dimension of financial inclusion are highly correlated. For example, a country with a high number of ATMs also tends to have more bank outlets. For that reason, we apply the principal component analysis (PCA) to our four financial inclusion indicators. The prerequisite condition for optimizing the estimates using the PCA requires the data to be Gaussian-distributed. As such, all indicators are standardized. We subtract the means from them and divide the results by their variance, as presented in Eq. (1) below.

(1) $$D_{i}=\frac{x_{i}-{\bar{x}}_{i}}{\sigma_{i}^{2}}$$

where i=1,2,3,4; denotes four indicators, including (i) the number of ATMs per 100,000 adults, (ii) the number of commercial bank outlets per 100,000 adults, (iii) the number of credit cards per 1,000 adults and (iv) the number of debit card per 1,000 adults. $\sigma_{i}^{2}$ and ${\bar{x}}_{i}$ are correspondingly variance and mean value of the indicator i.

The first principal component represents the linear combination of four constituent vectors, which explain the most redundancy presented through collecting data. The other components are similar to the first component. However, they are ordered from the largest to the smallest in the magnitude of variation. However, the choice of the number of components is controversial and varies across studies. For example, Ahamed and Mallick (2019) only considered the values whose eigenvalues are more significant than 1. In this paper, we follow the works of Abrahamsen and Hansen (2011), He et al. (2017), Vo et al. (2020), and Zhao and Yang (2020) by choosing the 85 per cent level of noise explanations for the borderline. The progress through which we construct our financial inclusion index is represented in Eqs. (2) and (3) as follows.

(2) $$PC_{m}=\sum_{k=1}^{4}D_{k}\times {Loading}_{k}$$

(3) $${IFI}_{i,t}=\sum_{m=1}^{M_{i}}PC_{m}\times IVC_{m}$$

where $D_{k};k=1,2,3,4$ are standardized financial inclusion indicators. $Loading_{k}$ stands for the factor loadings of the corresponding indicator k. $PC_{m}$ denotes the m^th^ principal component. $IVC_{m}$ stands for the individual variance contribution of m^th^ principal components. The $M_{i}$ is the number of components which we use to calculate the index of financial inclusion for country i, given that the cumulative individual variance contribution (IVC) has to exceed 85 per cent.

Table 2 presents the financial inclusion index (IFI) by a country level and its mean and standard deviation.

**Table 2:** The index of financial inclusion (IFI) for 19 countries in the Asia-Pacific region.

Country	IFI	Country	IFI	Country	IFI
Australia	0.717	Korea	0.445	Philippine	0.215
	(0.047)		(0.059)		(0.006)
Cambodia	0.047	Laos	0.020	Singapore	0.645
	(0.024)		(0.013)		(0.058)
China	0.534	Malaysia	0.434	Solomon	0.182
	(0.031)		(0.059)		(0.025)
Fiji	0.237	Maldives	0.233	Thailand	0.310
	(0.020)		(0.014)		(0.128)
India	0.259	Mongolia	0.628	Vietnam	0.129
	(0.026)		(0.063)		(0.053)
Indonesia	0.119	Nepal	0.055		
	(0.052)		(0.033)		
Japan	0.913	Pakistan	0.181		
	(0.043)		(0.003)		

Notes: The means of the index of financial inclusion (**IFI**) are presented at the country level. Standard deviation is reported in parentheses.

We note that countries that belong to the high-income group tend to have the financial sector more inclusive than countries belonging to the low-income group. Our index of financial inclusion estimates is quite similar to the index developed by Sarma (2008), Ahamed and Mallick (2019). Remarkably, countries generally considered a financial centre in the region have achieved a higher level of financial inclusion, such as Australia, Japan, and Singapore. In Asia, there is a wide range of IFI across countries since the continent itself is the largest one with various cultures and landscapes.

### 3.2 Methodology and data

#### 3.2.1 Data and model specification

Institutional quality refers to the degree to which the financial institutions extensively and intensively condition the financial development. In our study, the following five proxies are used in our analysis, as presented in Table 3. These choices are consistent with previous analyses (Nguyen et al., 2021; Jahan et al., 2019).

**Table 3:** The proxies for institutional quality and the data sources.

Proxy	The aspect of institutional quality	Data sources
*Business sophistication*	Capturing the regulatory efficiency	Heritage.org, a World governance indicator
*Regulatory quality*	Capturing the regulatory efficiency	Heritage.org, a World governance indicator
*Investment freedom*	Capturing the degree of financial liberalization	Heritage.org
*Government effectiveness*	Capturing the quality of public services such as education, healthcare, road, mobile telephony, and public transportation.	World Governance Indicator
*The rule of law*	Capturing the extent to which the economic agents are confident and compliant with the rules of society, contract enforcement, property rights, police, and the courts	World Governance Indicator

The determinants of financial inclusion are expressed in Eq. (4) as follows:

(4) IFI=f(Institution,Macroeconomics,Socialcharacteristics)

where IFI stands for our index of financial inclusion. *Institution* denotes the quality of governance in terms of (i) business sophistication, (ii) regulatory quality, (iii) investment freedom, (iv) government effectiveness, and (v) the rule of law. Macroeconomics includes the level of income, which is proxied by GDP per capita, and inflation .Finally,socialcharacteristics are proxied by the ratio between urban and total populations, denoted as population. Table 4 below presents the descriptive statistic of all included variables.

**Table 4:** The descriptive statistics for all variables.

Country	IFI	INF	GDPP	POP	BSF	REQ	EFL	GOE	ROL
Australia	0.72	2.30	10.82	4.45	4.50	1.79	4.35	1.68	1.76
	(0.05)	(0.88)	(0.24)	(0.01)	(0.03)	(0.1)	(0.06)	(0.12)	(0.06)
Cambodia	0.05	5.15	6.81	3.05	3.64	-0.49	4.02	-0.80	-1.08
	(0.03)	(1.63)	(0.64)	(0.13)	(0.12)	(0.06)	(0.15)	(0.22)	(0.15)
China	0.53	2.68	8.54	3.94	3.95	-0.22	3.27	0.22	-0.41
	(0.02)	(2.35)	(0.22)	(0.05)	(0.06)	(0.2)	(0.3)	(0.41)	(0.34)
Fiji	0.24	3.12	8.43	3.97	4.18	-0.46	3.64	-0.34	-0.40
	(0.03)	(2.95)	(0.42)	(0.07)	(0.12)	(0.2)	(0.16)	(0.23)	(0.17)
India	0.12	6.75	7.19	3.46	3.86	-0.33	3.66	0.02	0.01
	(0.05)	(2.83)	(0.37)	(0.06)	(0.19)	(0.1)	(0.15)	(0.17)	(0.08)
Indonesia	0.26	5.89	7.92	3.93	3.98	-0.28	3.57	-0.15	-0.53
	(0.04)	(0.9)	(0.1)	(0.03)	(0.07)	(0.12)	(0.12)	(0.15)	(0.12)
Japan	0.91	0.28	10.59	4.50	4.41	1.21	4.15	1.56	1.41
	(0.02)	(5.87)	(0.42)	(0.08)	(0.2)	(0.08)	(0.09)	(0.17)	(0.11)
Korea	0.44	2.14	10.14	4.40	4.47	0.96	4.24	1.14	1.03
	(0.06)	(1.24)	(0.21)	(0.003)	(0.09)	(0.14)	(0.02)	(0.13)	(0.11)
Lao PDR	0.02	4.65	7.19	3.44	4.03	-0.96	3.42	-0.77	-0.92
	(0.01)	(2.86)	(0.62)	(0.1)	(0.15)	(0.21)	(0.12)	(0.23)	(0.12)
Malaysia	0.43	2.13	9.09	4.27	4.35	0.61	3.80	1.04	0.50
	(0.01)	(4.36)	(0.31)	(0.06)	(0.05)	(0.31)	(0.11)	(0.21)	(0.23)
Maldives	0.23	3.70	8.86	3.61	4.42	-0.26	3.49	-0.21	-0.37
	(0.06)	(5.97)	(0.57)	(0.04)	(0.08)	(0.14)	(0.12)	(0.16)	(0.1)
Mongolia	0.63	9.14	7.89	4.20	4.21	-0.24	3.95	-0.43	-0.29
	(0.06)	(1.52)	(0.27)	(0.05)	(0.12)	(0.15)	(0.27)	(0.12)	(0.08)
Nepal	0.06	7.07	6.50	2.86	4.10	-0.70	2.53	-0.89	-0.70
	(0.03)	(2.71)	(0.48)	(0.1)	(0.06)	(0.11)	(0.70)	(0.09)	(0.14)
Pakistan	0.18	8.89	7.00	3.57	4.20	-0.64	3.79	-0.65	-0.81
	(0.003)	(4.30)	(0.23)	(0.03)	(0.1)	(0.09)	(0.22)	(0.14)	(0.09)
Philippines	0.22	3.83	7.75	3.83	4.01	-0.10	3.81	0.02	-0.46
	(0.01)	(1.89)	(0.36)	(0.01)	(0.1)	(0.1)	(0.29)	(0.1)	(0.09)
Singapore	0.64	1.69	10.78	4.61	4.57	1.97	4.41	2.21	1.73
	(0.06)	(2.07)	(0.28)	(0)	(0.03)	(0.2)	(0.07)	(0.12)	(0.11)
Solomon Islands	0.18	5.35	7.46	3.04	4.19	-1.03	2.60	-0.98	-0.51
	(0.02)	(4.48)	(0.34)	(0.11)	(0.04)	(0.15)	(0.23)	(0.14)	(0.23)
Thailand	0.31	1.99	8.54	3.80	4.30	0.21	3.71	0.31	-0.07
	(0.13)	(2.00)	(0.32)	(0.11)	(0.05)	(0.08)	(0.24)	(0.07)	(0.12)
Vietnam	0.13	7.39	7.32	3.46	4.07	-0.52	3.16	-0.14	-0.34
	(0.05)	(5.76)	(0.51)	(0.11)	(0.15)	(0.15)	(0.33)	(0.17)	(0.24)

Notes: The descriptive statistics of all variables. Standard deviations are in parentheses. **IFI** represents the index of financial inclusion, **INF** denotes inflation level; **GDPPC** denotes GDP per capita; **POP** represents the population which is measured by the ratio between urban population and total population; **BSF** denotes the business sophistication, **REQ** represents regulatory quality, **EFL** stands for the extent of investment freedom; **GOE** denotes the government effectiveness, **ROL** is proxied for the rule of law.

#### 3.2.2 The panel smooth transition regression

Our model, in general, is mathematically described in Eq. (5) as:

(5) $${IFI}_{it}=\beta_{0}+\beta_{1}institution_{it}+\beta_{2}X_{it}+\varepsilon_{it}$$

where $IFI_{it}$ is the financial inclusion index of country i in time t, $institution_{it}$ are proxied by five different proxies for institutional quality; $X_{it}$ represents the control variables for socio-economic factors, including *inflation*, *GDP per capita* and *Population*. $\varepsilon_{it}$ denotes other factors which jointly determine the level of financial inclusion.

We argue that the effects of institutional quality on financial inclusion in the Asia-Pacific region are asymmetric. Economic growth and income level have been found to affect financial inclusion. Countries in the Asia-Pacific region have exhibited significant differences in income levels. In the region, many countries are considered developed nations (Australia, Japan, Korea, Singapore), whereas others are still emerging markets (Thailand, Vietnam and many others in the region). In addition, regulations of the financial sectors are considered significantly different. Advanced countries have benefited from a well-established regulatory framework which is yet developed in most emerging markets. This paper is motivated by a significant difference in the income level and the current institutional framework. The panel smooth transition regression (PSTR) is used to separate the income effect from the effects of institutional quality on financial inclusion for countries in the Asia-Pacific region.

In a panel regression, the heterogeneous/individual effects might be detrimental to the unbiasedness of the estimators. These effects are even strengthened as the number of investigated countries becomes large. Since our panel data is considered large, the model with a homogeneous coefficient is inappropriate. Current literature indicates that there are several methods for addressing the problem of heterogeneity. Among these methods, the panel threshold regression (PTR) developed by Hansen (1999) is generally considered the advanced method. The method is based on the intuition that one control variable divides the sample into different regimes. The mechanism is similar to quantile regression. However, the panel smooth transition regression (PSTR) is superior to the traditional PTR. The method was first introduced in a seminal work by Gonzalez et al. (2005). The advantages of the PSTR are summarized as follows. *First*, the PSTR exhibits a transition phase by allowing each country to receive its corresponding slope based on its corresponding threshold variable on the bounded function $f\left(q_{it},\gamma ,c\right)$. *Second*, the function also stipulates the transition speed by the parameter γ. In sum, the PSTR is the regime-switching model, which defines the finite number of regimes and differentiates the heterogeneity of the slope parameters corresponding to each of them (Collezta and Hurlin, 2006).

## 4 Empirical findings

### 4.1 Tests

Since the PSTR estimator does not consider the dynamic change in the dependent variable, the index of financial inclusion, pre-tests are conducted to ensure the robustness of our estimation results. The first test examines the linear relationship between variables. The correlation matrix and the variance inflation factor (VIF) are reported in Table 5 below.

**Table 5:** The pairwise correlation and VIF for all variables.

Variables	VIF	GDPPC	POP	INF	BSF	REQ	EFL	GOE	ROL
GDPPC	7.86	1.000							
POP	6.90	0.869∗	1.000						
INF	1.49	-0.516∗	-0.388∗	1.000					
BSF	2.86	0.736∗	0.650∗	-0.318∗	1.000				
REQ	8.89	0.891∗	0.822∗	-0.441∗	0.668∗	1.000			
EFL	2.42	0.570∗	0.676∗	-0.271∗	0.342∗	0.687∗	1.000		
GOE	7.87	0.892∗	0.845∗	-0.468∗	0.644∗	0.960∗	0.631∗	1.000	
ROL	7.52	0.891∗	0.794∗	-0.429∗	0.707∗	0.946∗	0.582∗	0.944∗	1.000

Notes: ∗ denotes the statistical significance at 0.01 per cent level. **GDPPC** denotes GDP per capita; **POP** denotes the urban population - the ratio between urban population and total population; **INF** denotes inflation level **BSF** denotes the degree of business sophistication, **REQ** represents regulatory quality, **EFL** stands for the extent of investment freedom; **GOE** denotes the government effectiveness, **ROL** is proxied for the rule of law.

Table 5 indicates that none of the variables has a VIF greater than 10, meaning that no significant evidence of the existence of multi-co-linearity is found.

### 4.2 Empirical results

The effect of institutional quality on financial inclusion is considered asymmetric and conditional on the income level. We now use the Gonzalez et al. (2004) test to validate this consideration. Table 6 reports the results of the slope homogeneity test using the Pesaran and Yamagata (2008) test and the linearity test using the Gonzalez et al. (2005) test.

**Table 6:** Empirical results from the slope homogeneity test and the linearity test.

	Pesaran and Yamagata test				
	Business sophistication	Regulatory quality	Investment freedom	Government effectiveness	The rule of law
Δ	6.791∗∗∗	0.784	-0.353	10.490∗∗∗	2.422∗∗
	(0.000)	0.433	(0.724)	(0.000)	(0.015)
**Linearity test (H**_**0**_**: r = 0, H**_**a**_**: r = 1)**					
Lagrange Multiplier	35.922	40.139	35.421	37.362	41.444
	(0.000)	(0.000)	(0.000)	(0.000)	(0.000)
Lagrange Multiplier (Fisher-test)	9.749	11.206	9.581	10.237	11.674
	(0.000)	(0.000)	(0.000)	(0.000)	(0.000)
LRT Likelihood ratio test	39.890	45.183	39.270	41.680	46.852
	(0.000)	(0.000)	(0.000)	(0.000)	(0.000)
**Linearity test (H**_**0**_**: r = 1, H**_**a**_**: r = 2)**					
Larange Multiplier	16.256	17.483	15.674	11.247	15.468
	(0.003)	(0.002)	(0.003)	(0.024)	(0.004)
Larange Multiplier (Fisher-test)	3.713	4.022	3.568	2.496	3.517
	(0.000)	(0.004)	(0.008)	(0.045)	(0.009)
LRT Likelihood ratio test	17.007	18.355	16.370	11.600	16.146
	(0.000)	(0.001)	(0.003)	(0.021)	(0.003)
**Linearity test (H**_**0**_**: r = 2, H**_**a**_**: r = 3)**					
Larange Multiplier	6.498	10.005	14.771		5.568
	(0.165)	(0.040)	(0.005)		(0.234)
Larange Multiplier (Fisher-test)	1.368	2.148	3.259		1.168
	(0.248)	(0.078)	(0.013)		(0.328)
LRT Likelihood ratio test	6.613	10.283	15.387		5.653
	(0.158)	(0.036)	(0.004)		(0.227)

Notes: ∗, ∗∗, ∗∗∗, respectively denotes the statistical significance at 10, 5, 1 percent level. P-values are reported in parentheses. All possible versions of the test statistic are reported. The null hypothesis of Pesaran and Yamagata (2008) is that the slope coefficients are homogeneous. **BSF** denotes the degree of business sophistication, **REQ** denotes the regulatory quality, **EFL** captures investment freedom, **GOE** is proxied for government effectiveness, and **ROL** denotes the rule of law.

Table 6 confirms that both tests reject the assumption of homogenous coefficients across our sample. Moreover, results from the Gonzalez test confirm that our sample should be divided into three distinct regimes concerning the impacts of the income effects on financial inclusion. Accordingly, we now categorize 19 countries in our sample into three groups based on the estimated parameters from the non-linear regression. These three groups of countries include (i) the low-income countries whose average income is below 5,000 USD/person/year; (ii) the middle-income countries whose average income ranges from 5,000to22,000 USD/person/year; and (iii) the high-income countries whose average income is from 22,000 USD/person/year and greater. the estimated results using the PSTR estimator for five different proxies for institutional quality. Key findings from our analyses can be summarized as follows.

*First*, we find a positive relationship between inflation and financial inclusion in high-income countries. As inflation increases by one per cent, financial inclusion increases by 0.02 per cent. This finding implies more outstanding debt at financial institutions and more people with debit or credit accounts. The inflation rate is commonly seen as a lag economic indicator for economic growth. Countries with reasonable monetary policies can keep the inflation rate under control to attract foreign and domestic capital.

*Second*, concerning economic growth, which is also our threshold variable, we observe that the middle-income countries appear to have a higher degree of financial inclusion than the lower-income countries. However, a similar pattern cannot be established between the high-income countries and the middle-income countries. Our findings imply that financial inclusion improves the overall income level in the Asia-Pacific region, particularly in low-income countries. The empirical results from our PSTR are similar to findings from previous studies such as Van et al. (2019), Kim et al. (2017), and Cihak et al. (2016). Our findings also support the conclusions of Sarma and Pais (2011) and Levine et al. (2000) on the heterogeneous effects of socio-economic factors on financial inclusion.

*Third*, urbanization/domestic migration is closely linked with financial inclusion. When more people move to the cities seeking better economic opportunities, the degree of financial inclusion is higher. This is because people work and adapt to urban livelihood. They will then contribute positively not only to economic growth but also to financial inclusion. Our sample also indicates the difference in the extent of financial inclusion between rural and urban areas in many countries. However, the effects of urbanization on financial inclusion in high-income countries are significantly lower than those in low-income countries and middle-income countries.

Results from Table 7 demonstrate that this effect reduces as the average income per capita increases. This finding is supported by the negative signs of the estimated coefficients in the other two regimes. In detail, a one per cent increase in urban population is associated with a 1.06 per cent increase in financial inclusion of the low-income countries (for business sophistication), 0.98 per cent (for investment freedom), 1.04 per cent (for the rule of law), 1.07 per cent and 1.2 per cent (for government effectiveness and regulatory quality). These estimated coefficients, which are greater than one, demonstrate that urbanization has a spillover effect on other underdeveloped regions of the country. We consider that the migrants to the cities appear to engage in the financial system. They may also influence their relatives who live in rural areas to use financial products.

**Table 7:** The coefficients, transitive speeds and extreme values using the PSTR estimator.

Independent variable	Coefficients	Proxies for institutional quality				
		Model 1	Model 2	Model 3	Model 4	Model 5
**Inflation** (INF)	$\beta_{1}$	-0.0001	0.0002	-0.001		-0.001
	(low-income)	(-0.2805)	(0.351)	(-1.086)		(-1.069)
	$\beta_{2}$	-0.001	-0.003∗	-0.002	0.0011	-1.95e-5
	(middle-income)	(-0.317)	(-1.907)	(-0.367)	(1.472)	(-0.009)
	$\beta_{3}$	-0.017∗∗∗	0.021∗∗∗	0.038∗∗∗	0.0165∗∗∗	0.017∗∗∗
	(high-income)	(4.326)	(4.889)	(4.884)	(4.285)	(3.735)
**Income level** (GDPPC)	$\beta_{1}$	-0.014	-0.044∗	-0.066∗∗∗		-0.050∗∗
	(low-income)	(-0.783)	(-1.725)	(-2.637)		(-2.161)
	$\beta_{2}$	0.085∗∗∗	0.1607∗∗∗	-0.127∗∗∗	-0.0072	0.142∗∗∗
	(middle-income)	(3.826)	(4.625)	(-3.239)	(-0.368)	(4.248)
	$\beta_{3}$	0.0057	-0.041	-0.150	0.078∗∗	-0.008
	(high-income)	(0.1937)	(-1.204)	(-1.59)	(2.523)	(-0.220)
**Urban population** (POP)	$\beta_{1}$	1.058∗∗∗	0.979∗∗∗	1.038∗∗∗		1.171∗∗∗
	(low-income)	(13.410)	(9.403)	(11.730)		(12.479)
	$\beta_{2}$	-0.089	-0.389∗∗∗	0.341∗∗∗	1.0727∗∗∗	-0. 321∗∗∗
	(middle-income)	(-1.366)	(-5.166)	(3.807)	(13.389)	(-4.223)
	$\beta_{3}$	-0.378∗∗∗	0.065	0.255	-0.221∗∗∗	-0.0135
	(high-income)	(-4.022)	(0.0842)	(1.156)	(-2.951)	(-0.178)
**Business sophistication**	$\beta_{1}$	-0.039∗∗				
	(low-income)	(-2.198)				
	$\beta_{2}$	-0.106∗∗∗				
	(middle-income)	(-4.014)				
	$\beta_{3}$	0.329∗∗∗				
	(high-income)	(3.775)				
**Regulatory quality**	$\beta_{1}$		-0.135∗∗∗			
	(low-income)		(-7.077)			
	$\beta_{2}$		0.151∗∗∗			
	(middle-income)		(-4.538)			
	$\beta_{3}$		-0.463			
	(high-income)		(-1.052)			
**Investment freedom**	$\beta_{1}$			-0.04∗∗∗		
	(low-income)			(-4.549)		
	$\beta_{2}$			0.052∗∗∗		
	(middle-income)			(2.68)		
	$\beta_{3}$			-0.016		
	(high-income)			(-0.266)		
**Government effectiveness**	$\beta_{1}$				-0.057∗∗∗	
	(low-income)				(-3.709)	
	$\beta_{2}$				0.0371	
	(middle-income)				(0.977)	
	$\beta_{3}$					
	(high-income)					
**The rule of law**	$\beta_{1}$					-0.062∗∗∗
	(low-income)					(-3.543)
	$\beta_{2}$					-0.168∗∗∗
	(middle-income)					(-4.007)
	$\beta_{3}$					0.443∗∗∗
	(high-income)					(5.264)
Trans. speed 1	$\gamma_{1}$	14.746	6.086	16.835	75.142	4.027
Center of mass 1	$c_{1}$	7.727	8.068	8.364	10.147	8.937
Trans. speed 2	$\gamma_{2}$	49.090	29.399	23.888		4.113
Center of mass 2	$c_{2}$	10.137	10.119	10.116		10.666

Notes: T-statistics are reported in parentheses. ∗, ∗∗, ∗∗∗, respectively denote the statistical significance at 10 per cent, 5 per cent, and 1 per cent levels.

We now shift our focus to the asymmetric effect of institutional quality on the degree of financial inclusion concerning each proxy for institutional quality. Our empirical findings confirm that high-income countries, in general, dominate the other two groups of countries concerning the degree of financial inclusion. Concerning regulatory quality and business freedom, governments should implement policies and regulations that encourage the growth of the private sector. Opportunities for business activities mean an increase in newly registered firms and, as a result, greater economic expansions across countries. Specifically, as the opportunity for private sector development increases by one per cent, countries with an average income of greater than 22,000 USD will benefit from an increase of 0.25 per cent of newly opened accounts. This number of new accounts opening represents the number of jobs that the private sectors have created for people who migrate to the urban areas.

We also find a positive effect from the regulatory quality and the rule of law on financial inclusion for high-income countries. We consider that judicial fairness significantly reduces the legal risks in terms of dispute or a failure to comply with the host countries' law that might apply to the local business operations. For particular countries, the cost associated with the mitigation of legal risks, such as the cost of legal consultants, can be exorbitantly high. As a result, these countries' opportunities for doing business might become less attractive and economically infeasible than in other countries.

## 5 Conclusions and policy implications

The effects of financial inclusion on economic growth have increasingly attracted attention from scholars and governments in recent years, especially for emerging markets. As a result, various studies have been conducted to investigate the effect. Previous studies document the positive effects of financial inclusion on multiple aspects of society and the economy, such as income inequality, economic growth, and financial stability. However, the effects of institutional quality on financial inclusion have largely been ignored in the existing literature, particularly the asymmetrical effects. As such, this study examines the asymmetrical effects of the institutional quality on financial inclusion to shed light on the channels which the governments can consider to promote all-inclusive Finance. We use the annual financial inclusion data from the financial access surveys (FAS) to estimate the financial inclusion index for 19 Asia-Pacific countries. We also incorporate the time-variant and time-invariant heterogeneity between each country in the sample when investigating the asymmetrical effects of institutional quality on financial inclusion using the panel smooth transition regression.

Key findings from our study can be summarized as follows. First, the institutional quality provides asymmetric effects on financial inclusion depending on different socio-economic circumstances across countries in the sample.

Our empirical results confirm the positive relationship between inflation and financial inclusion in high-income countries. The middle-income countries are found to exhibit a higher degree of financial inclusion than lower-income countries. However, a similar pattern cannot be established between high-income and middle-income countries within our research sample and period. Urbanization is also found to affect financial inclusion. We find a significant difference in the degree of financial inclusion between rural and urban areas in many countries in our sample. We also find that the effects of urbanization on financial inclusion in high-income countries are significantly lower than those in low-income countries and middle-income countries.

Interestingly, the effects of institutional quality on financial inclusion are different for different countries. Our empirical evidence confirms a positive effect for high-income countries and an adverse effect for low-income countries. However, the effects of institutional quality on financial inclusion are mixed for middle-income countries.

We consider that financial markets develop in a fragile manner without adequate corporate governance. Therefore, it is appropriate for low-income or middle-income countries to initiate and implement policies on developing the financial markets and products. In detail, the private sectors should actively participate in providing inputs into the policymaking process, aiming to achieve the desired policies for both private and public sectors.

Policy implications have emerged based on the findings of this paper. For emerging markets such as Vietnam and other middle-income countries in the Asia-Pacific region, improving institutional quality appears to improve and enhance financial inclusion – the desired policy to support sustainable economic growth and development in the near future. Improving institutional quality requires the governments to formulate and implement policies to improve judicial independence and legal enforcement to ensure property rights and the successful deregulation of the financial markets. In addition, flexibility in the national monetary policy is desirable. It allows the central banks to set targets for inflation, control the money supply satisfactorily, and allow citizens to open accounts in foreign currencies.

In addition, the current product and service distribution network are still mainly based on the traditional way of organization, through branches, transaction offices and transaction points. Modern service delivery channels via mobile devices and the Internet, such as debit cards, credit cards, ATMs, Internet Banking, Mobile Banking, and SMS Banking, have not been fully utilized and exploited to their full potential. However, in Vietnam, the percentage of people using mobile phones and accessing the internet is among the highest in the world.

For payment infrastructure, the development of an automatic electronic clearing system for low-value retail transactions has been slow, reducing the efficiency of inter-bank retail payment processing. Services and means of personal payment are not genuinely convenient. Payment infrastructure such as POS/ATM systems is unevenly developed. This infrastructure is located mainly in urban areas. The investment in this type of infrastructure is significant. Regarding information and telecommunications infrastructure, a national database on the population has been unavailable, hindering financial institutions from looking up and retrieving information. People lacking information will struggle to access credit services.

The government needs to accelerate the construction of infrastructure for the financial sector. Mobilizing social capital or ODA for infrastructure development programs is a reasonable goal with a tight budget. In addition, the regulator also needs to support the banking system to expand coverage by utilizing factors with substantial coverage across the country as access points.

Our study exhibits limitations. We consider that future studies on this important issue of financial inclusion should consider the following notes. *First*, this paper selects various indicators, such as the number of debit and credit cards representing a degree of financial inclusion. It is argued that individuals can hold these cards and never use them. As such, other indicators, such as the deposits and credits with and from formal financial institutions, appear to be appropriate for use as the proxies for financial inclusion. *Second,* it is complicated to measure a true degree of financial inclusion. The existing literature indicates that various approaches to estimating the degree of financial inclusion have been initiated and used in various empirical analyses, such as Camara and Tuesta (2014), Ahamed and Mallik (2019), Tram et al. (2021), Avom et al. (2021) and many others. Future studies will need to conduct a thorough investigation of these different approaches to compare and contrast the strengths and weaknesses.

## Declarations

### Author contribution statement

Loan Thi-Hong Van; Nhan Thien Nguyen; Hung Le-Phuc Nguyen; Duc Hong Vo: Conceived and designed the experiments; Performed the experiments; Analyzed and interpreted the data; Contributed reagents, materials, analysis tools or data; Wrote the paper.

### Funding statement

This research is funded by Ho Chi Minh City Open University under the grant number E2021.04.1 (Loan Van).

### Data availability statement

Data will be made available on request.

### Declaration of interests statement

The authors declare no conflict of interest.

### Additional information

No additional information is available for this paper.
